# Analysis of Epstein–Barr Virus (EBV) and PD-L1 Expression in Nasopharyngeal Carcinoma Patients in a Non-Endemic Region

**DOI:** 10.3390/ijms231911720

**Published:** 2022-10-03

**Authors:** Josiane M. Dias, Iara V. V. Santana, Vinicius D. da Silva, André L. Carvalho, Lidia M. R. B. Arantes

**Affiliations:** 1 Clinical Oncology Department, Barretos Cancer Hospital, Barretos 14784-400, SP, Brazil; 2 Pathology Department, Barretos Cancer Hospital, Barretos 14784-400, SP, Brazil; 3 Molecular Oncology Research Center, Barretos Cancer Hospital, Barretos 14784-400, SP, Brazil; 4 Early Detection, Prevention & Infections Branch, International Agency for Research on Cancer, 69372 Lyon, France

**Keywords:** nasopharyngeal carcinoma, Epstein–Barr virus (EBV), programmed cell death ligand-1 (PD-L1), non-endemic region

## Abstract

Background: The purpose of this study was to evaluate the status of Epstein–Barr virus (EBV) infection and the expression of programmed cell death ligand-1 (PD-L1) in tumor samples from patients with nasopharyngeal carcinoma (NPC). Methods: Evaluation of EBV infection was performed through the detection of EBV-encoded small ribonucleic acids (EBER) by in situ hybridization, and PD-L1 expression was performed through immunohistochemistry. Results: In total, 124 samples were evaluated for EBER and 120 for PD-L1 expression. A total of 86.3% of cases were positive for EBER and 55.8% were positive for PD-L1. There was a correlation between EBER positivity and the presence of undifferentiated carcinoma histology (*p* = 0.007) as well as the absence of tobacco history (*p* = 0.019). There was a correlation between PD-L1 expression and EBER positivity (*p* = 0.004). There was no statistically significant difference between overall survival (OS) and EBER (*p* = 0.290) or PD-L1 (*p* = 0.801) expression. Conclusions: This study corresponds to one of the largest cohorts of NPC in a non-endemic region. Phase III studies with checkpoint inhibitors are ongoing and may provide more data about the role of PD-L1 expression in this disease.

## 1. Introduction

Nasopharyngeal carcinoma (NPC) comprises the majority of cancers that arise in the nasopharynx in populations with high and low incidence of the disease. The World Health Organization (WHO) classifies NPC in three histological types: keratinizing squamous cell carcinoma (KSCC), non-keratinizing squamous cell carcinoma (NKSCC—includes undifferentiated carcinoma) and basaloid squamous cell carcinoma (BSCC) [1].

NPC is endemic in some areas of Southeast Asia, Polynesia, East Asia and North Africa, but it is rare in the Western world [2]. In the United States and Western Europe, the incidence is 0.5 to 2 cases per 100,000 persons. In southern China, the incidence reaches 25 cases per 100,000 inhabitants per year [3]. In Brazil, it is a rare neoplasm but it caused 374 deaths in 2019 and 28% of these deaths occurred in individuals under 50 years [4].

The frequency of each histological type varies between endemic regions and the Western countries. Data from the United States Surveillance, Epidemiology and End Results Program (SEER) show that KSCC accounted for 39.4% of cases, while undifferentiated carcinoma (UC) accounted for 25% of the total [5]. In Southern China, 95% of the NPC related to UC, while only 2% comprise KSCC and 3% NKSCC [6].

The distinct geographic distribution suggests a multifactorial etiology for the disease. In low-incidence areas, NPC is associated with alcohol consumption and smoking [7]. In high-incidence regions, the risk seems to depend on the interaction of several factors such as Epstein–Barr virus (EBV) infection, genetic predisposition and lifestyle habits [8].

EBV infection is present in almost all cases of UC in endemic areas, but it is less frequent in KSCC. Some evidence supports the association of EBV in the etiology of NPC, such as the detection of viral deoxyribonucleic acid (DNA) in tumor cells, as well as the expression of viral antigens in the neoplastic cell membrane [9].

In infected tumor cells, there is expression of multiple EBV antigens, such as EBV-encoded small ribonucleic acids (EBERs), that can block cell apoptosis. Detection of EBER by in situ hybridization (ISH) is a practical method to detect the presence of EBV in NPC tumor cells [10].

Treatment is defined according to the clinical stage and patient’s clinical condition. It is based on radiotherapy alone or in association with platinum-based chemotherapy, whether induction, concomitant, adjuvant or palliative [11]. With the advent of immunotherapy, the action of checkpoint inhibitors such as anti-PD-1 (anti-programmed death-1) and anti-PD-L1 (anti-programmed death-ligand 1) has been increasingly studied in NPC [12].

EBV infection causes expression of viral proteins in tumor cells such as latent membrane protein 1 (LMP1) in addition to the activation of interferon-gamma (IFN-γ). Both can synergistically induce PD-L1 expression in NPC and some reports have associated high PD-L1 expression with worse clinical outcomes [13,14,15].

In the Brazilian population, there are few data regarding the prevalence of EBV in NPC, as well as no reports about the expression of PD-L1 in this population [16,17]. Therefore, this study evaluated the status of EBV infection and PD-L1 expression in tumor samples from patients with NPC treated in Brazil, in addition to the correlation with histological type and clinical-demographic and survival characteristics.

## 2. Results

### 2.1. Clinical-Demographic Characteristics

Of the 139 patients with NPC who underwent screening, 124 were eligible for this study. Among these cases, all were evaluated for EBER and 120 samples were submitted to PD-L1 analysis (4 cases did not have enough material). All 124 samples were positive for vimentin.

Among the 124 cases analyzed, 77.4% were male and the median age was 51 years (ranging from 17 to 88 years), and 46.7% were 50 years or less at diagnosis. No age peaks were observed. Most cases were from the southeast region of Brazil (65.3%), with no history of smoking (56.7%) or alcoholism (59.5%). The predominant histological type was non-keratinizing SCC (96%) and most cases (53.2%) had locally advanced disease (EC IVa) (Table 1).

### 2.2. EBER Analysis

Regarding EBER analysis, 86.3% of the cases were positive (representative image in Supplementary Figure S1A,B). Among the 72 undifferentiated carcinoma samples, 98.6% were positive for EBER. There was a statistically significant correlation between EBER positivity and the following variables: positive expression of PD-L1 (*p* = 0.004), absence of alcoholism (*p* = 0.028), absence of tobacco history (*p* = 0.001), absence of comorbidity (*p* = 0.022) and UC histology (*p* < 0.001) (Table 2). After adjusting the model through multivariate analysis, only undifferentiated carcinoma histology maintained the correlation with EBER positivity (OR = 18.72; 95% CI 2.25–155.86; *p* = 0.007) as well as absence of smoking history (OR = 7.15; 95% CI 1.38–36.92; *p* = 0.019).

### 2.3. PD-L1 Analysis

Among the 120 samples analyzed for PD-L1 expression, 55.8% were positive (representative image in Supplementary Figure S1C,D). In 25.8% of these samples, there was high expression of tumor proportion score (TPS) (value ≥ 50%) and in 34.2% of cases the combined positive score (CPS) was ≥20 (Table 3). There was a statistically significant correlation between PD-L1 expression and positivity for EBER (*p* = 0.004) (Table 4). After adjusting the model through multivariate analysis, this significant correlation was maintained (OR = 5.11; 95% CI 1.55–16.80; *p* = 0.007). There was no correlation between PD-L1 expression and any clinical-demographic variable.

**Figure 1:**
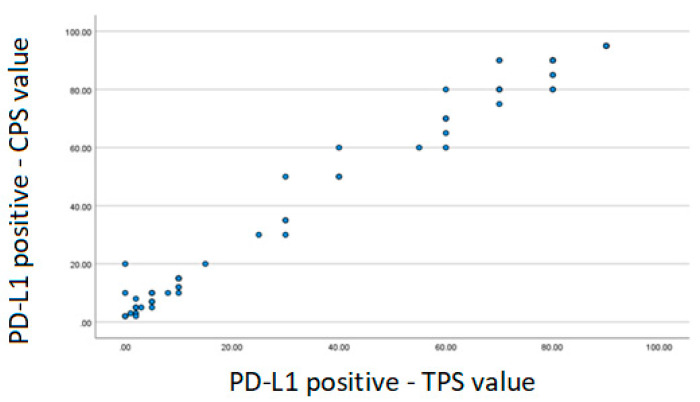
Scatter plot—Spearman correlation. PD-L1: programmed cell death ligand-1; TPS: tumor proportion score; CPS: combined positive score.

Spearman’s correlation was performed between the positive TPS and CPS variables. There was a strong correlation demonstrated through the coefficient value of 0.969 (Figure 1).

### 2.4. Survival Analysis

Survival analyses were performed in 122 cases (1 case was excluded due to loss of follow-up during the initial treatment and 1 case was discarded due to the patient’s death before start of treatment). The median follow-up time was 46.15 months (ranging from 1.38 to 130.69 months).

**Figure 2:**
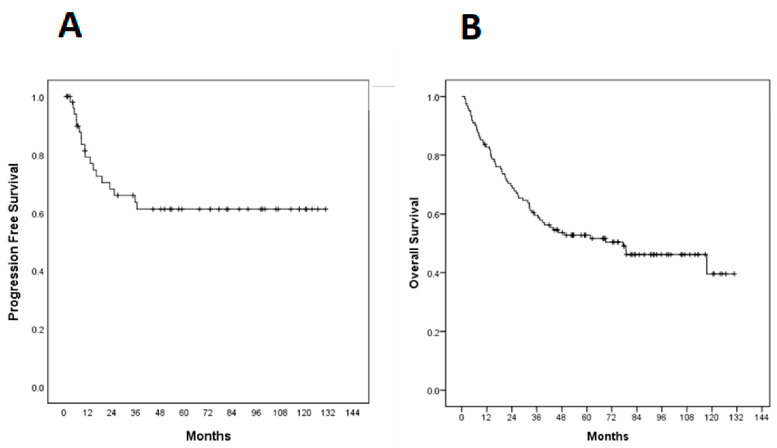
Kaplan–Meier curves in 122 cases. (**A**) Progression-free survival (PFS); (**B**) Overall survival (OS).

**Figure 3:**
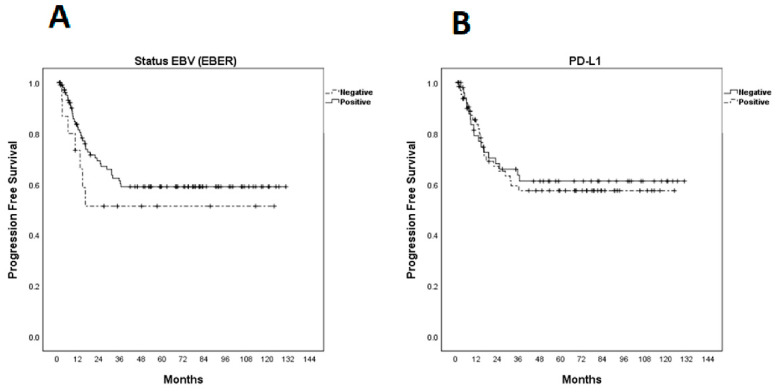
Kaplan–Meier curves for progression-free survival (PFS). (**A**) PFS according to EBER (Epstein–Barr-virus-encoded small RNAs); (**B**) PFS according to programmed cell death ligand-1 expression.

Median progression-free survival (PFS) had not yet been achieved at the time of analysis and was estimated to be 59.9% at 3 years (Figure 2). There was no statistically significant difference between PFS and EBER expression (*p* = 0.336) or PD-L1 (*p* = 0.772) (Figure 3).

**Figure 4:**
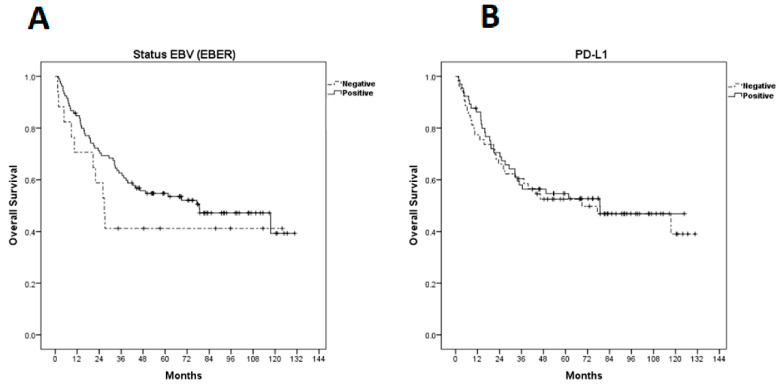
Kaplan–Meier curves for overall survival (OS). (**A**) OS according to EBER (Epstein–Barr-virus-encoded small RNAs); (**B**) OS according to programmed cell death ligand-1 expression.

The median overall survival (OS) was 77.4 months (95% CI 33.57–121.22 months) and estimated 5-year OS was 52.7% (Figure 2). There was no statistically significant difference between OS and EBER expression (*p* = 0.290) or PD-L1 (*p* = 0.801) (Figure 4).

Additionally, there was no significant difference regarding OS when comparing patients with high PD-L1 expression (TPS ≥ 50%) versus low expression (TPS < 50%) or negative TPS (*p* = 0.750) or when compared in relation to high CPS expression (≥20) versus low CPS expression (˂20) or negative CPS (*p* = 0.846).

## 3. Discussion

This is one of the largest series with NPC carriers in a non-endemic region and the first study that described the analysis of PD-L1 in NPC in the Brazilian population.

In this study, we observed a high prevalence of the non-keratinizing SCC subtype, reported in 96% of the sample. The keratinizing subtype was rare, only seen in 0.8% of the cases. These results differ from other series from non-endemic regions. Ruuskanen and colleagues reported in a Finnish population that 78% of the cases corresponded to non-keratinizing SCC and 22% to keratinizing SCC [18]. Moreover, Ou and colleagues reviewed data from 2640 patients from the United States using the SEER database and found that the keratinizing subtype accounted for 39.4% of the cases [5].

It is interesting to note that Breda and colleagues, when reviewing data from 320 patients with NPC in Portugal, found that 95.75% of the cases corresponded to the non-keratinizing SCC, similar to our series [19]. Brazil was colonized by Portugal and one hypothesis is that this colonization may be related to the similar results with the Portuguese population.

Another relevant fact from our study was the high positivity for EBER among the undifferentiated carcinoma samples, which corresponded to 98.6%. This high prevalence is seen in populations from endemic regions, where studies demonstrate up to 100% EBV expression in undifferentiated NPC [20,21].

Regarding the expression of PD-L1, this study found 55.8% positivity. The analysis was performed for both TPS and CPS. A strong correlation was observed between them when we performed Spearman’s correlation. Thus, when PD-L1 was positive, there was an association between the value of TPS and CPS. Note that the higher the TPS value, the higher the CPS value.

In other tumor sites related to head and neck cancer such as oropharynx, larynx, hypopharynx and oral cavity, the decision regarding the use of immunotherapy for the treatment of advanced disease takes into account the value of CPS [22]. This analysis evidenced this strong correlation between CPS and TPS and it is hypothesized that in NPC only the TPS assessment can be carried out. The TPS analysis is simpler than the CPS and the pathologist’s analysis time could be optimized.

A positive point of this study was the analysis of vimentin through immunohistochemistry prior to PD-L1 analysis. All samples were positive for vimentin. The purpose of this evaluation was to try to assess the immunoantigenicity of the samples to avoid false-negative cases for PD-L1, since some cases originated from biopsies performed in an external service. The vimentin is uniformly distributed in tissues and it serves as an ideal internal quality control of tissue fixation [23].

In a multivariate analysis, a positive correlation was observed between EBER expression and PD-L1 positivity. Some studies have reported the occurrence of PD-L1 overexpression in EBV-related NPC. Fang and colleagues proposed two possible mechanisms for this overexpression: the first would be involved with the adaptive immune system, in which PD-L1 would be induced in response to inflammatory signals such as IFN-γ that would be produced in an antiviral immune response. The second mechanism would be related to the innate immune system, in which the activation of the constitutive oncogenic pathway mediated by LMP1 generates the overexpression of PD-L1 [14].

Regarding this second mechanism, Fang and colleagues showed that LMP1 induced PD-L1 expression through Jak3/Stat3, Mapks/AP-1 and P65/NF-κB pathways in human NPC cells [14]. Although we understand the importance of evaluating LMP1 expression in this study, it was not feasible due to the limited amount of FFPE tissue.

This study showed no difference in OS or PFS according to EBER or PD-L1 expression. Literature data show that EBER positivity is associated with a better prognosis, with better survival rates. Kengjian and colleagues analyzed overall survival in 908 NPC patients and showed that EBER expression was an independent risk factor for overall survival [24].

The literature data are controversial about PD-L1 expression and prognostic factors. Some series show a better prognosis for high PD-L1 expression, while other studies show worse survival rates [25,26,27,28]. Ma and colleagues reported in a phase II trial using nivolumab in metastatic and/or refractory NPC that 33% of PD-L1-positive patients responded to nivolumab, while only 13% of those with PD-L1 negative tumors responded; however, this did not reach statistical significance. This lack of statistical correlation could be due to the limited sample size [29]. On the other hand, the randomized phase III trial JUPITER-02 that evaluated toripalimab or placebo in combination with cisplatin and gemcitabine in recurrent or metastatic NPC did not observe any difference in PFS between subgroups with different PD-L1 expression [30].

One of the limitations of the current study is that it is a retrospective analysis and was carried out in a single institution. Most of the patients were born and came from the Southeast and Midwest regions of Brazil and the other country regions were poorly represented in this cohort.

## 4. Materials and Methods

This was a retrospective and observational cohort study conducted at Barretos Cancer Hospital, Brazil. One hundred and thirty-nine patients had histologically confirmed NPC between January 2010 and December 2017 and were identified through institution’s database. All samples were reviewed by an experienced pathologist to confirm the diagnosis and characterize the cellular components of the specimen. Samples that raised doubts were discussed together with a second pathologist and both defined the final result. Patient demographic, clinical characteristics, treatments and outcomes were recorded from medical records. Clinical stage was according to eighth edition of the American Joint Committee on Cancer (AJCC) staging of NPC.

### 4.1. Inclusion Criteria

Patients with tumor located in nasopharynx, confirmed diagnosis of carcinoma, biopsy performed before starting the treatment and tumor samples available for analysis were included.

### 4.2. Exclusion Criteria

Anatomopathological results consistent with a different histological type of carcinoma and previously treated patients were excluded.

### 4.3. EBER Analysis

Hematoxylin-and-eosin-stained sections of 3–4 µm thickness were prepared after retrieving the formalin-fixed paraffin-embedded (FFPE) tissue blocks from the histopathology laboratory. EBV status was assessed by EBER detection through in situ hybridization (ISH) using the Ventana^®^ ISH iView Blue Detection Kit (Roche^®^ Diagnostics GmbH, Germany) and automated BenchMarck^®^ Ultra staining module according to manufacturer’s instructions (Ventana^®^ Medical Systems Inc., United States). EBER status was classified as positive or negative, as defined by the presence or absence of deep dark blue signal in nuclei of tumor cells seen under light microscopy.

### 4.4. PD-L1 Expression

The PD-L1 expression was determined by immunohistochemical reaction using the Benchmark^®^ ULTRA platform and the anti-PD-L1 rabbit monoclonal antibody (Cell Signaling Technology^®^, United States, clone E1L3N and dilution 1:200) for detection of PD-L1 protein using the Optiview DAB^®^ visualization system. This process was performed according to manufacturer’s specifications and the samples were analyzed by two pathologists with specialized training to evaluate the expression of PD-L1. A minimum of 100 viable tumor cells must have been present for the specimen to be considered evaluable.

PD-L1 expression was determined through two scores: tumor proportion score (TPS) and combined positive score (CPS). TPS was determined by the percentage of viable tumor cells that showed partial or complete staining of the membrane in relation to all viable tumor cells present in the sample. It was graded on a scale of 0% to 100% and was positive if detected in at least 1% of viable tumor cells.

The CPS was calculated using the number of positive PD-L1 stained cells (sum of tumor cells, lymphocytes and macrophages) divided by the total number of viable tumor cells in the slide, multiplied by 100. It was scored as negative if less than 1 and positive if greater than or equal to 1. Positive cases were graded on a scale of 1 to 100.

### 4.5. Vimentin Analysis

Analysis of vimentin expression in tumor tissue was performed by immunohistochemical reaction. The purpose of this analysis was to carry out an internal control of the fixation’s quality and processing of the tissue to be analyzed. It is known that during the formaldehyde fixation process, irregular preservation of antigens in tumor sample may occur. This can generate false-negative results in immunohistochemical analysis such as PD-L1.

Vimentin is a molecule evenly distributed in tissue samples such as vessels and stromal cells. Thus, an antibody that detects a vimentin epitope is suitable for controlling the sample fixation process.

The vimentin analysis was performed by immunohistochemical reaction. The automation platform BenchMark Ventana Ultra^®^ and the mouse monoclonal anti-vimentin antibody (clone V9) from Ventana Roche^®^ were used. The detection system used was the Ultraview DAB^®^. The entire process was carried out according to the manufacturer’s specifications.

The reading of the reaction was performed using an optical microscope by the pathologist. The absence of expression was classified as negative. The reaction was evaluated as positive when there was any expression of vimentin, in cytoplasm or membrane, either in tumor cells or in the stroma of the sample. Thus, negative cases for vimentin expression were excluded from the analysis.

### 4.6. Statistical Analysis

The results of the EBER and PD-L1 analysis were correlated with the patients’ clinical and pathological data, using the statistical program SPSS (Statistical Package for the Social Sciences) 23.0 for Windows. Analysis was performed using the chi-square and Fisher’s exact tests to verify the association between anatomopathological variables and molecular results. Survival rates were assessed using the Kaplan–Meier method and the curves were compared using the log-rank test. Statistical significance was determined for a *p* value < 0.05.

## 5. Conclusions

This study corresponds to one of the largest series of NPC in a non-endemic region. The predominant histological profile was non-keratinizing squamous cell carcinoma, showing similarity with endemic regions.

There was a correlation between EBER positivity and the presence of undifferentiated carcinoma histology as well as the absence of tobacco history. PD-L1 expression was positive in just over half of the cases. There was a correlation between PD-L1 expression and EBER positivity.

Phase III studies with checkpoint inhibitors are currently underway in NPC patients and may provide more data about the role of PD-L1 expression in this disease.

## Figures and Tables

**Table 1:** Clinical-demographic characteristics.

Variable	*n* = 124
Median age, years (range)	51 (17–88)
Gender Female Male	28 (22.6%) 96 (77.4%)
Race/skin color White Brown/Black	95 (78.5%) 26 (21.5%)
Origin Southeast region Midwest region Other regions	81 (65.3%) 33 (26.6%) 10 (8.1%)
Smoking habits Never Active Former smoker	64 (56.7%) 24 (21.2%) 25 (22.1%)
Alcoholism habits No Yes	69 (59.5%) 47 (40.5%)
Biopsy site Primary tumor Cervical lymph node	87 (70.2%) 37 (29.8%)
Histology Keratinizing SCC * Differentiated non-keratinizing SCC Undifferentiated non-keratinizing SCC Basaloid SCC	01 (0.8%) 47 (37.9%) 72 (58.1%) 04 (3.2%)
Clinical Stage ** I II III IVa IVb	02 (1.6%) 08 (6.5%) 31 (25.2%) 66 (53.7%) 16 (13.0%)
ECOG PS *** 0 1 2 3	15 (12.1%) 95 (76.6%) 12 (9.7%) 02 (1.6%)

* SCC: squamous cell carcinoma; ** AJCC: American Joint Committee on Cancer (8th edition); *** ECOG PS: Eastern Cooperative Group Performance Status.

**Table 2:** Univariate analysis for EBER * positivity.

Variable	EBER Positive	EBER Negative	*p* Value
Gender Male Female	*n* (%) 83 (77.6%) 24 (22.4%)	*n* (%) 13 (76.5%) 4 (23.5%)	0.920
Race/Skin color White Brown/Black	83 (79.0%) 22 (21.0%)	12 (75.0%) 4 (25.0%)	0.746
Clinical Stage I II III IVa IVb	2 (1.9%) 7 (6.6%) 27 (25.5%) 56 (52.8%) 14 (13.2%)	0 (0%) 1 (5.9%) 4 (23.5%) 10 (58.8%) 2 (11.8%)	0.976
PD-L1 ** Positive Negative	63 (61.2%) 40 (38.8%)	4 (23.5%) 13 (76.5%)	0.004
Alcoholism Habits No Yes	63 (63.6%) 36 (36.4%)	6 (35.3%) 11 (64.7%)	0.028
Smoking Habits Never Acive/Former smoker	61 (62.9%) 36 (37.1%)	3 (18.7%) 13 (81.3%)	0.001
Comorbidity Absent Present	69 (64.5%) 38 (35.5%)	6 (35.3%) 11 (64.7%)	0.022
Non-keratinizing SCC ^1^ Undifferentiated Differentiated	71 (67.0%) 35 (33.0%)	1 (7.7%) 12 (92.3%)	<0.001

* EBER: Epstein–Barr-virus-encoded small RNAs; ** PD-L1: programmed cell death ligand-1; ^1^ SCC: squamous cell carcinoma.

**Table 3:** Analysis of EBER * and PD-L1 **.

EBER (*n* = 124) Positive Negative	*n* (%) 107 (86.3) 17 (13.7)
PD-L1 (*n* = 120) Positive Negative	*n* (%) 67 (55.8) 53 (44.2)
PD-L1—analysis of TPS ^1^ Negative Positive 1–49% Positive ≥ 50%	*n* (%) 58 (48.4) 31 (25.8) 31 (25.8)
PD-L1—analysis of CPS ^2^ Negative Positive 1–19 Positive ≥ 20	*n* (%) 53 (44.2) 26 (21.6) 41 (34.2)

* EBER: Epstein–Barr-virus-encoded small RNAs; ** PD-L1: programmed cell death ligand-1; ^1^ TPS: tumor proportion score; ^2^ CPS: combined positive score.

**Table 4:** Univariate analysis for PD-L1 * positivity.

Variable	PD-L1 Positive	PD-L1 Negative	*p* Value
Gender Male Female	*n* (%) 50 (74.6%) 17 (26.4%)	*n* (%) 43 (81.1%) 10 (18.9%)	0.397
Race/Skin color White Brown/Black	50 (76.9%) 15 (23.1%)	41 (78.8%) 11 (21.2%)	0.804
Clinical Stage I II III IVa IVb	1 (1.5%) 5 (7.5%) 18 (26.9%) 34 (50.7%) 9 (13.4%)	1 (1.9%) 2 (3.8%) 13 (25.0%) 29 (55.8%) 7 (13.5%)	0.930
EBER ** Positive Negative	63 (61.2%) 40 (38.8%)	4 (23.5%) 13 (76.5%)	0.004
Alcoholism Habits No Yes	39 (61.9%) 24 (38.1%)	27 (55.1%) 22 (44.9%)	0.468
Smoking Habits Never Active/Former smoker	40 (63.5%) 23 (36.5%)	21 (45.7%) 25 (54.3%)	0.064
Comorbidity Absent Present	44 (65.7%) 23 (34.3%)	29 (54.7%) 24 (45.3%)	0.222
Non-keratinizing SCC ^1^ Undifferentiated Differentiated	45 (67.2%) 22 (32.8%)	26 (54.2%) 22 (45.8%)	0.157

* PD-L1: programmed cell death ligand-1; ** EBER: Epstein–Barr-virus-encoded small RNAs; ^1^ SCC: squamous cell carcinoma.

## Data Availability

Not applicable.

## Supplementary Materials

The following supporting information can be downloaded at: https://www.mdpi.com/article/10.3390/ijms231911720/s1.
