# Supplementary material for: Analysis of Epstein–Barr Virus (EBV) and PD-L1 Expression in Nasopharyngeal Carcinoma Patients in a Non-Endemic Region
Source: Int J Mol Sci. 2022 Oct 3;23(19):11720. doi: 10.3390/ijms231911720 (PMC9569432; doi:10.3390/ijms231911720)
Supplement: Supplementary file 1 [file ijms-23-11720-s001.zip › ijms-1894643-supplementary.pptx]

## Slide 1
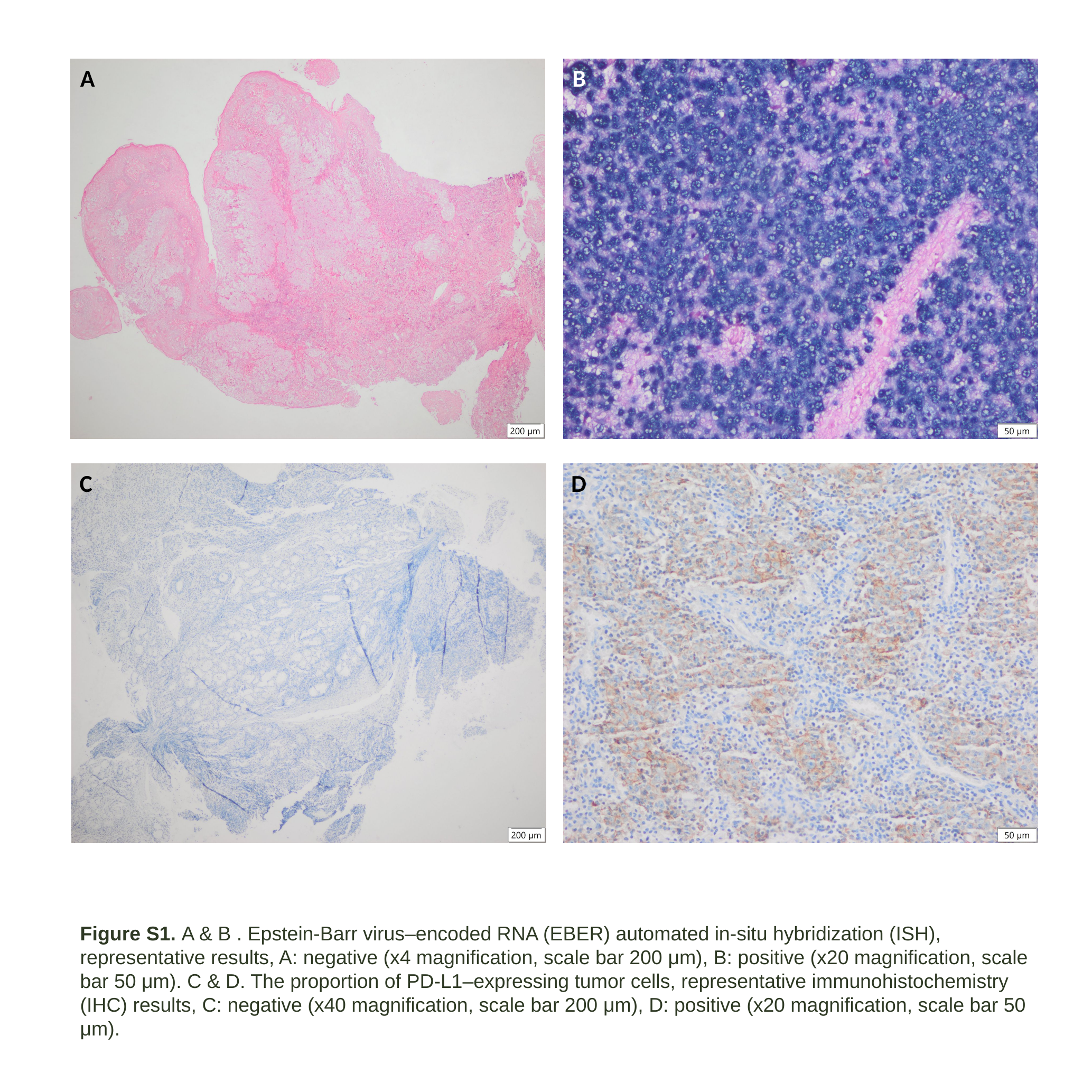

A
B
C
D
Figure S1. A & B . Epstein-Barr virus–encoded RNA (EBER) automated in-situ hybridization (ISH), representative results, A: negative (x4 magnification, scale bar 200 μm), B: positive (x20 magnification, scale bar 50 μm). C & D. The proportion of PD-L1–expressing tumor cells, representative immunohistochemistry (IHC) results, C: negative (x40 magnification, scale bar 200 μm), D: positive (x20 magnification, scale bar 50 μm).
